# Directed-evolution mutations enhance DNA-binding affinity and protein stability of the adenine base editor ABE8e

**DOI:** 10.1007/s00018-024-05263-7

**Published:** 2024-06-14

**Authors:** Haixia Zhu, Lei Wang, Ying Wang, Xinyi Jiang, Qin Qin, Menghua Song, Qiang Huang

**Affiliations:** 1 State Key Laboratory of Genetic Engineering, Shanghai Engineering Research Center of Industrial Microorganisms, MOE Engineering Research Center of Gene Technology, School of Life Sciences, Fudan University, Shanghai, 200438 China; 2 Multiscale Research Institute of Complex Systems, Fudan University, Shanghai, 201203 China

**Keywords:** Genome editing, Base editor, ABE8e, DNA binding, Electrostatic interactions, Thermal stability

## Abstract

Adenine base editors (ABEs), consisting of CRISPR Cas nickase and deaminase, can chemically convert the A:T base pair to G:C. ABE8e, an evolved variant of the base editor ABE7.10, contains eight directed evolution mutations in its deaminase TadA8e that significantly increase its base editing activity. However, the functional implications of these mutations remain unclear. Here, we combined molecular dynamics (MD) simulations and experimental measurements to investigate the role of the directed-evolution mutations in the base editing catalysis. MD simulations showed that the DNA-binding affinity of TadA8e is higher than that of the original deaminase TadA7.10 in ABE7.10 and is mainly driven by electrostatic interactions. The directed-evolution mutations increase the positive charge density in the DNA-binding region, thereby enhancing the electrostatic attraction of TadA8e to DNA. We identified R111, N119 and N167 as the key mutations for the enhanced DNA binding and confirmed them by microscale thermophoresis (MST) and in vivo reversion mutation experiments. Unexpectedly, we also found that the directed mutations improved the thermal stability of TadA8e by ~ 12 °C (T_m_, melting temperature) and that of ABE8e by ~ 9 °C, respectively. Our results demonstrate that the directed-evolution mutations improve the substrate-binding ability and protein stability of ABE8e, thus providing a rational basis for further editing optimisation of the system.

**Supplementary Information:**

The online version contains supplementary material available at 10.1007/s00018-024-05263-7.

## Introduction

CRISPR (clustered regularly interspaced short palindromic repeats) systems have been widely used as molecular machines for targeted genome editing [1–5]. In general, Cas (CRISPR-associated) nucleases induce double-stranded breaks (DSBs) at target DNA sites, which are repaired by non-homologous end joining (NHEJ) and homology-directed repair (HDR), ultimately leading to genome modification [2, 6, 7]. However, NHEJ is an uncontrollable pathway that usually results in random insertion or deletion mutations (indels) [8, 9]. And the addition of a donor DNA template can stimulate HDR to precisely modify the gene, but this pathway occurs with low efficiency [10, 11]. Thus, although the CRISPR system is an efficient tool for chemically disrupting genes, applications that modify bases at specific DNA sites require more precise DNA editing tools [12–14]. For example, the largest class of known human pathogenic mutations are point mutations, the correction of which requires very precise site-specific editing [15, 16].

**Fig. 1:**
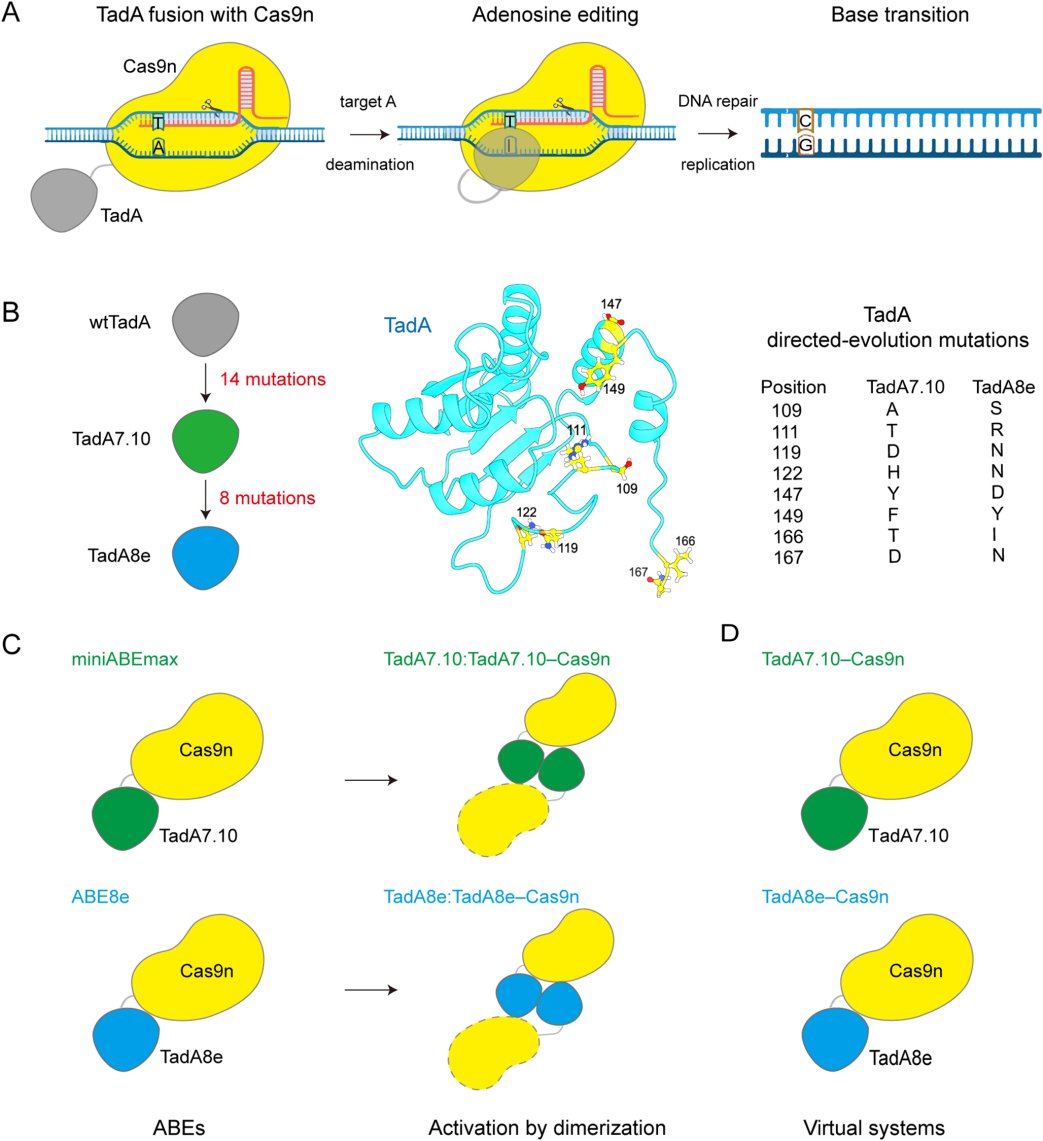
Overview of adenine base editing. (**A**) Schematic representation of how ABE acts on DNA. (**B**) The directed evolution process of TadA8e and the eight directed-evolution mutations in TadA8e that lead to increased editing activity of ABE8e. (**C**) Two realistically functioning ABE systems constructed for the simulations. (**D**) Two virtual ABE systems constructed for the simulation comparison with the systems in C

To avoid unwanted mutations, DNA base editors (BEs) have been developed to allow programmable conversion of the target base pairs without creating DSBs [17–19]. BEs consist of a Cas9 nickase (Cas9n) and a deaminase that chemically acts on single-stranded DNA (ssDNA). After the single guide RNA (sgRNA) is paired with the DNA target strand (TS), a segment of the DNA non-target strand (NTS) becomes unpaired, and the DNA bases within this ssDNA segment are modified by the deaminase; at the same time, the Cas nickase cleaves the unedited TS, inducing cells to use the edited NTS as a template to repair the TS, and ultimately accomplish the base editing (Fig. 1A) [17, 18, 20]. To date, two types of BEs have been discovered: cytosine base editors (CBEs), which convert a C: G base pair into T: A [17, 21], and adenine base editors (ABEs), which convert an A: T base pair to G: C [18, 22]. It is estimated that approximate 60% of human disease-associated point mutations can be corrected by these two BEs. And ABE, in particular, can correct up to 47% of the mutations [15, 16].

To develop ABEs, Liu and co-workers first used directed evolution to screen for variants of fusion proteins consisting of *E. coli* tRNA adenosine deaminase (TadA) and Cas9n, and successfully obtained an active BE called ABE7.10 (Fig. 1B) [18]. ABE7.10 contains two TadAs: the wild-type tRNA deaminase TadA (wtTadA) and the evolved deaminase TadA7.10 from wtTadA, and these two TadAs are fused to the SpCas9 nickase as wtTadA‒TadA7.10‒Cas9n. It has been shown that in the absence of wtTadA, the catalytic (editing) activity of the so-called miniABEmax (i.e., TadA7.10‒Cas9n) is reduced (Fig. 1C) [18, 23, 24]. In order to increase its activity and compatibility with other Cas effectors, additional rounds of directed evolution were performed and then eight mutations were introduced into TadA7.10, resulting in a more active BE with the newly evolved TadA called TadA8e (Fig. 1B), namely ABE8e (Fig. 1C) [22]. Compared to ABE7.10, ABE8e requires only one deaminase (TadA8e), but has the same editing activity as in the presence of wtTadA. In vitro, ABE8e deaminates DNA at a rate 590- and 1170-fold higher than ABE7.10 and miniABEmax, respectively and its editing level at the same target sites was 3 to 11 times higher than that of ABE7.10. Thus, the eight directed-evolution mutations in TadA8e of ABE8e significantly improve the catalytic activity [22, 23].

As a rational basis, it is important to understand the mechanistic and functional roles of the directed-evolution mutations in the base editing catalysis of ABEs in order to optimise them for more efficient and precise editing by protein engineering. To this end, Rallapalli et al. have shown that the initial mutations reduce the structural flexibility and lead to conformational changes in the early-evolved TadA [25]. Lapinate et al. have determined the first cryo-electron microscopy (cryo-EM) structure of ABE8e (PDB ID: 6VPC), and showed that the directed-evolution mutations could stabilise the DNA substrate in a constrained, transfer RNA-like conformation. Meanwhile, they found that TadA8e of ABE8e exists as a homodimer [23]. Indeed, in *E. coli* wtTadA functions as a homodimer, with one monomer catalysing deamination and the other monomer contributing to the tRNA-substrate binding [26]. Since none of the directed-evolution mutations in TadA7.10 and TadA8e are located in their dimerization interfaces, it is likely that the evolved ABE variants reported to date retain their ability to dimerise [23]. In other words, miniABEmax and ABE8e could also function in their dimerization forms as Cas9n‒TadA7.10:TadA7.10‒Cas9n and Cas9n‒TadA8e: TadA8e‒Cas9n, respectively. Therefore, despite many efforts, the functional implications of the eight directed-evolution mutations in TadA8e of ABE8e remain unclear.

Here, we combined molecular dynamics (MD) simulations and experimental measurements to elucidate the chemical roles or effects of the eight directed-evolution mutations in TadA8e. Our MD simulations showed that the DNA-binding affinity of TadA8e was approximately 1.9 ~ 8.9 times that of TadA7.10, and electrostatic interactions were the major driving force. The directed-evolution mutations significantly increased the positive charge density in the DNA-binding region, thereby enhancing the electrostatic attraction between TadA8e and the single-stranded DNA substrate. In addition, we found that the directed-evolution mutations also improved the thermal stability of TadA8e and ABE8e, with a melting temperature (T_m_) increase of 12 °C and 9 °C, respectively. Consequently, these results highlight the functional roles of the DNA binding and protein stability in the base editing catalysis of ABEs.

## Materials and methods

### Construction of full-length ABE models

The cryo-EM structure of ABE8e (PDB ID: 6VPC) was used as the major template to construct all the full-length models of ABEs for the MD simulations. The missing atoms and loops of the ABE proteins were constructed using homology modeling at SWISS-MODEL (https://swissmodel.expasy.org). The TadA7.10 structure was generated by introducing point mutations into the TadA8e structure using the mutagenesis plugin in PyMOL. The NTS of PDB ID 5F9R was used as a template to complement the missing NTS bases in 6VPC.The DNA building process was as follows: firstly, the sgRNA-DNA backbone of the 5F9R structure was superimposed onto that of 6VPC. Next, A_12_-A_19_ in the NTS strand of the superimposed 5F9R were grafted to the corresponding missing C_31_-G_38_ positions in the NTS strand of 6VPC, and then this grafted NTS was mutated from the sequence of 5F9R to that of 6VPC by using the mutagenesis plugin in PyMOL. Finally, energy minimization was performed to optimize the structural model. Surface electrostatic potentials for TadA8e and TadA7.10 were calculated using the APBS, the protein dielectric was set to 4, and the solvent dielectric set 80 [27, 28]. All visualisations and interaction analyses were performed using ChimeraX [29, 30], GROMACS software [31, 32] and Python script.

### MD simulations and analyses

All MD simulations were performed using GROMACS. The topology and coordinate files were generated using the *pdb2gmx* program with parameters from the AMBER99BSC1 force field [33]. The complex was placed in the center of a cubic box with an edge length of ~ 15 Å from the protein surface to the box boundary, and then solvated with TIP3P water molecules [34]. Specific numbers of Na^+^ and Cl^−^ ions were added to the system to neutralize the complex charge and the ion concentration was set to 0.15 M. To optimise the system, energy minimisation was performed using the steepest descent algorithm for a maximum of 2500 steps or until the maximum force < 1000 kJ·mol^− 1^·nm^− 1^. The system was then heated to 310 K for 100 ps with a time step of 2 fs by constant NVT equilibration.

Constant NPT simulations of 500 ps were then performed to equilibrate the system at a pressure of 1.0 bar. The simulated temperature and pressure were maintained at 310 K and 1.0 bar using the V-rescale temperature and Parrinello–Rahman pressure coupling methods, respectively [35, 36]. During the simulations, all bonds were constrained using the LINCS method [37], and the periodic boundary conditions (PBC) were applied in all three dimensions. The short-range non-bonded interactions were calculated for the atom pairs within a 14 Å cut-off, while the long-range electrostatic interactions were calculated using the Particle Mesh Ewald (PME) method [38]. Finally, 500 ns production simulations were performed.

The free energy landscapes were generated using the *gmx_sham* command and plotted with RMSD and Rg as the projection components. The mapping method was based on the study of Jiang et al. [39].

### Binding energy calculations

The *g_mmpbsa* package was used to calculate the binding energy between the TadAs and NTS by the molecular mechanics Poisson–Boltzmann surface area (MM/PBSA) method [40, 41]. The energy components E_MM_, G_polar_, and G_nonpolar_ of each complex were calculated for the snapshots extracted from the MD production simulations from 150 to 500 ns (350 snapshots). The electrostatic (E_elec_), van der Waals (E_vdW_) interactions, and polar solvation energies are modeled using a Coulomb, Lennard-Jones (LJ) potential function and Poisson–Boltzmann (PB) equation, respectively. The G_polar_ was calculated by solving the nonlinear PB equation, and the nonpolar energy was calculated by the solvent-accessible surface area (SASA) method [42]. The dielectric constant of the solvent was set to 80, and four values (2, 4, 6 and 8) were used for the biomolecules [43]. The Python script *MmPbSaStat.py* was used for the MM-PBSA calculation and *MmPbSaDecomp.py* was used to calculate the contributions of individual residues in the TadA.

### Protein expression and purification

TadA7.10, TadA7.10-3mut and TadA8e, carrying an N-terminal non-cleavable His_6_-tag, were expressed in *E. coli* Rosetta (DE3). Cells were grown in LB containing 50 µg/mL kanamycin and 25 µg/mL chloramphenicol at 37 ℃ to an OD_600_ of approximately 1.0, and were induced with 0.5 mM IPTG. Growth was continued at 25 ℃ for 12 h. Cells were harvested by centrifugation and resuspended in buffer: 20 mM HEPES pH7.5, 500 mM KCl, 20 mM imidazole, 10% glycerol, 2 mM β-mercaptoethanol (β-ME) supplemented with protease inhibitor 1 mM PMSF, and lysed by sonication on ice. After centrifugation at 8,000 rpm for 1 h, the supernatant was loaded onto Poly-Prep chromatography columns (BIO-RAD) containing Ni-NTA agarose (QIAGEN), and proteins were eluted with a gradient of buffer supplemented with 500 mM imidazole. The eluted proteins were further purified by size exclusion chromatography using a Superdex 200 Increase 10/300 GL column developed in 20 mM HEPES pH7.5, 500 mM KCl buffer. Final purity was checked by the SDS-PAGE. Eluted proteins were flash-frozen in liquid nitrogen and stored at − 80 °C.

### Microscale thermophoresis (MST)

MST experiments to measure the binding affinities between TadAs and the 19-nt DNA substrate were performed on a Monolith NT.115 system (NanoTemper Technologies, Germany) using the nano BLUE detector. The MST experiments were performed with 15% LED-power and 40% IR-laser power. Laser on and off times were set to 30 s and 5 s, respectively. The 19-nt DNA labeled with 6-FAM at the 5’-end was purchased from Sangon Biotech Co., Ltd. (Shanghai, China). The final concentration of 19-nt DNA used was 100 nM, and TadAs were diluted in binding buffer (20 mM HEPES pH 7.5, 200 mM KCl, 5 mM MgCl_2_, 1 mM DTT, supplemented with 0.05% Tween-20) starting at 2000 nM at a ratio of 1:1 for 10 concentration gradients. Samples were incubated for 120 min at room temperature and then filled into capillaries (NanoTemper Technologies) for the measurements. Measurements were performed at least three times, and the resulting data were analysed using the MO.Affinity analysis software (NanoTemper Technologies).

### Thermal shift assay

The melting temperature T_m_ of TadA and ABE proteins were determined using nano-differential scanning fluorimetry (nanoDSF, Nanotemper Prometheus NT.48 system) [44]. The temperature was increased from 25 to 95 °C at a ramp rate of 1 °C/min. The excitation wavelength was set at 280 nm and the instrument monitored the emission fluorescence at 350 nm and 330 nm. In the measurements, the recorded ratio of the emission intensities (Em_350nm_/Em_330nm_) represents the change in tryptophan fluorescence. The T_m_ value for each experiment of the given TadA and ABE proteins was then automatically calculated using the PR.ThermControl software by plotting the ratiometric measurement of the fluorescence signal against the increasing temperature.

### Transfection of HEK293T cells and preparation of genomic DNA

HEK293T cells were seeded into 10 cm dish (WHB). 12–15 h after plating, cells were transfected with 30 µL of PEI (Maokang biotechnology) using 7.5 µg of base-editor plasmid with green fluorescent protein GFP tag and 2.5 µg of guide RNA plasmid. Cells were cultured for 3 d before washing with PBS (137.0 mM NaCl, 2.7 mM KCl, 10.0 mM Na_2_HPO_4_, 1.8 mM KH_2_PO_4_, pH 7.4). Flow cytometry analysis was carried out using a MA900 Multi-Application Cell Sorter (Sony). Genomic DNA was extracted using the TIANamp Genomic DNA Kit (TIANGEN) according to the manufacturer’s instructions. Protospacer sequences for guide RNA plasmids are described in Supplementary Table 1. Primers for PCR amplification of target genomic sites are listed in Supplementary Tables 2, and amplicons for analyses are listed in Supplementary Table 3.

## Results

### MD simulations of full-length ABEs

It is well established that enzymatic activity is regulated by many factors, including conformational dynamics and substrate-binding affinity [45–48]. Additionally, enzymatic stability also plays a key role in preserving enzymatic activity under varying environmental conditions [49]. To investigate the effects of the eight directed mutations on the conformational dynamics of TadA8e of ABE8e and its interaction with DNA, we first constructed two simulation systems for the real functional forms of ABE8e and ABE7.10 with additional, dimerized TadAs. The systems were designated as TadA8e: TadA8e‒Cas9n and TadA7.10:TadA7.10‒Cas9n, respectively (Fig. 1C, right panel). For comparison, two corresponding, virtual ABE systems without the dimerized TadAs were also constructed: TadA8e‒Cas9n and TadA7.10‒Cas9n, respectively (Fig. 1D).

To construct these systems, we first used the cryo-EM structure (PDB ID: 6VPC) as the protein template to build the full-length atomic model of TadA8e:TadA8e‒Cas9n. However, the 32-aa peptide linker between Cas9n and TadA8e and several NTS bases are missing in the cryo-EM structure. To complete the coordinates of these atoms, we compared the structure with all available Cas9‒sgRNA‒DNA ternary complexes in PDB to identify the active SpCas9 structures whose sgRNA‒DNA conformations are similar to that of 6VPC [50, 51]. Consequently, the NTS of the most similar structure (PDB ID: 5F9R) was employed as the template to complete the missing NTS bases in 6VPC (Fig. S1A). We next constructed the 32-aa peptide linker using SWISS-MODEL (https://swissmodel.expasy.org) and 6VPC as the protein template (Fig. S1B) and thus obtained the full-length model consisting of sgRNA, DNA, TadA8e: TadA8e‒Cas9n (Fig. S1C). It should be noted that one model was also predicted directly by AlphaFold2 with the amino acid sequence (Fig. S1B).However, this model was found to be significantly different from 6VPC and therefore not used. We introduced point mutations into the two TadA8e structures of TadA8e: TadA8e‒Cas9n using the *mutagenesis* plugin in PyMOL and then generated the full-length model for TadA7.10:TadA7.10‒Cas9n. By removing their dimerized, the models for TadA7.10‒Cas9n and TadA8e‒Cas9n were constructed. Finally, energy minimization was performed to refine the atomic coordinates of all four models.

Using the above full-length models, four simulation systems were constructed as described in Materials and Methods. Each system comprised approximately 630,000 atoms. To obtain equilibrated structures for the subsequent analyses, for each system all-atom MD simulations of approximately 500 ns were performed. To assess whether the systems were equilibrated, we calculated root mean square deviations (RMSDs) of the ABE backbone atoms using their initial structures as the references. As shown in Fig. S2, the RMSDs of the four systems showed no significant changes after about 150-ns simulations, indicating that the simulated ABE complexes were equilibrated. Therefore, the MD complex structures of the four systems after the 150-ns simulations were used for the analyses.

### The structure of TadA8e is more stable than that of TadA7.10

**Fig. 2:**
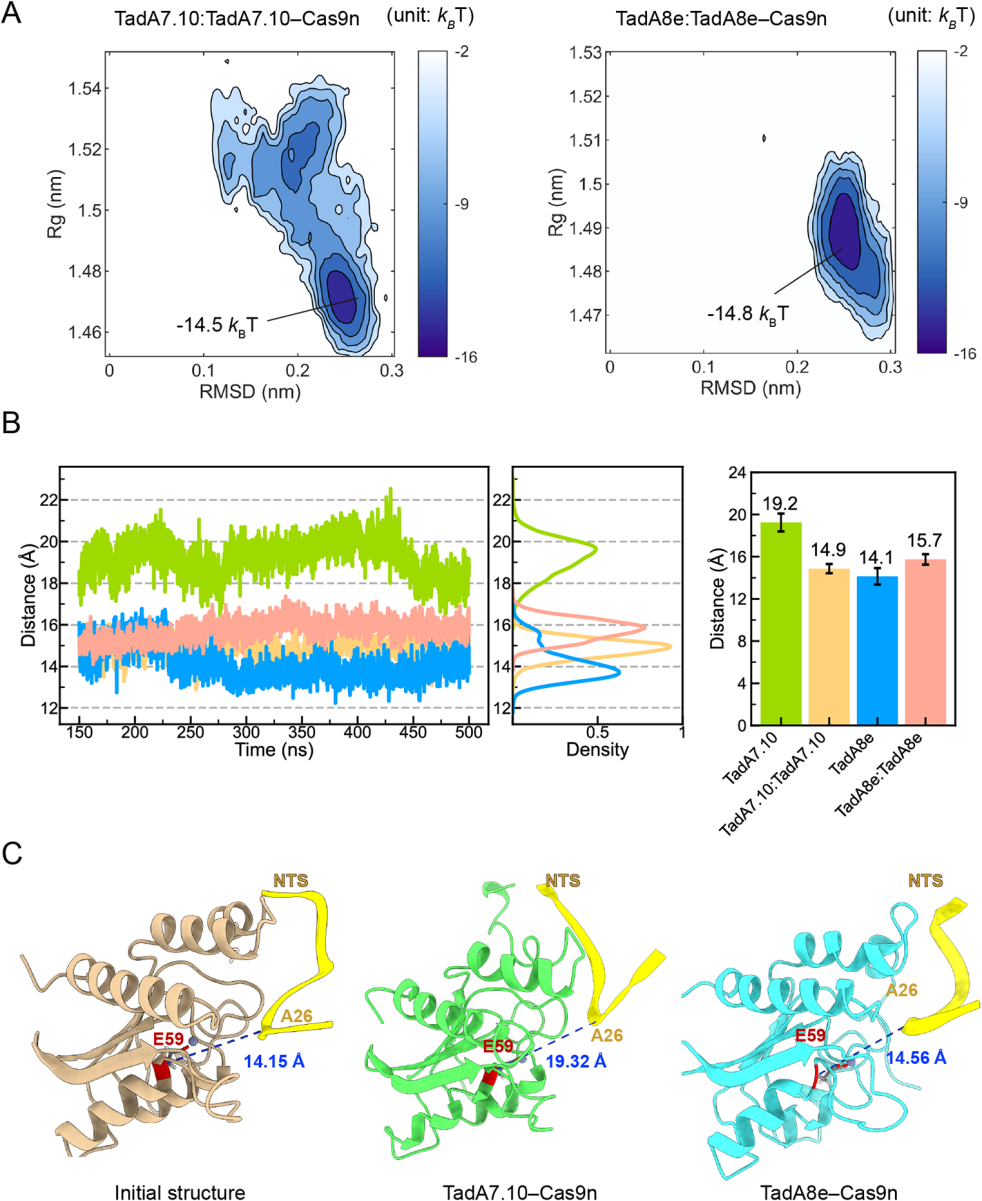
The conformational states of TadA7.10 and TadA8e in the MD simulations. (**A**) Free energy landscapes of TadAs projected onto RMSD of the protein backbone atoms and radius of gyration (Rg), where *k*_B_ is the Boltzmann constant and *T* is the simulation temperature (310 K). (**B**) Time-dependent distances and corresponding distributions of the Cα atom of TadA E59 at the active site to the C4’ atom of the substrate A26. (**C**) The initial and equilibrium conformations of TadA. The distances from Cα of TadA E59 to C4’ of the substrate A26 (blue dotted lines)

As shown in Fig. S1C, only the first TadA that was fused to Cas9n is responsible for the deamination, whereas the second, dimerized TadA does not directly interact with the DNA substrate [23]. Therefore, we mainly analysed the conformational dynamics of the Cas9n-fused TadA and its interactions with the DNA substrate in TadA8e: TadA8e‒Cas9n or TadA7.10:TadA7.10‒Cas9n. First, we investigated the conformational stability of the fused TadA7.10 and TadA8e by projecting their free energy landscapes onto two components: RMSD and radius of gyration (Rg). As shown in Fig. 2A, the fused TadA7.10 in TadA7.10:TadA7.10‒Cas9n possesses three deep energy wells, suggesting that TadA7.10 contains multiple dominant conformations. In contrast, the fused TadA8e in TaA8e: TadA8e‒Cas9n has only one deep well, suggesting that TadA8e has a single, more stable conformation than the fused TadA7.10. This suggests that the eight directed-evolution mutations enhance the conformational stability of TadA8e.

Similar to dimeric TadA8e, monomeric TadA8e in TadA8e‒Cas9n also has one deep well of -14.1 *k*_B_T, which is comparable to that of dimeric TadA8e (~ -14.8 *k*_B_T), indicating that the conformational stabilities of TadA8e are similar in both monomeric and dimeric forms (Fig. S3). In contrast, monomeric TadA7.10 in TadA7.10‒Cas9n has two deep wells of about -12.1 *k*_B_*T*, higher than that (-14.5 *k*_B_*T*) in the dimeric form, suggesting that the second TadA7.10 stabilises the first TadA7.10 fused to Cas9n. Again, the monomeric TadA7.10 without the eight directed-evolution mutations is a more flexible compared to TadA8e.

To explore the effects of the TadA structural stability on substrate recruitment, we calculated the distances between the residue E59 in the active sites of TadAs and base A26 of the DNA substrate (Fig. 2B). In TadA8e‒Cas9n, TadA8e: TadA8e‒Cas9n and TadA7.10:TadA7.10‒Cas9n, the average distances from the Cα atom of E59 to the C’4 atom of A26 were very similar at ~ 14.1 Å, ~ 15.7 Å and ~ 14.9 Å, respectively (Fig. 2B-C). However, the corresponding distance in TadA7.10‒Cas9n was up to 19.2 Å and significantly larger than that in TadA7.10:TadA7.10‒Cas9n (Fig. 2B-C). This suggests that the structural stability of TadAs is important for their binding to the DNA substrate, and that the dimeric form is required to stabilise the fused TadA7.10 for its tighter binding to the DNA substrate.

### TadA8e has a higher binding affinity for DNA than TadA7.10

To test the above hypothesis, we used the *g_mmpbsa* program [40] to calculate the energies for the TadA binding to the substrate DNA in the four systems. It is well known that the relative dielectric constant of the solute is a key parameter in the MM/PBSA calculation of *g_mmpbsa* [40, 43]. Considering that the investigated ABEs are protein-DNA systems, to rationalise the comparison, we used four different solute dielectric constants (i.e., 2, 4, 6 and 8) in the calculations, where the minimum 2 is commonly used for low-polarised proteins [43, 52], and the maximum 8 is the DNA dielectric constant [53]. The calculated binding energies of TadA to the ssDNA substrate (i.e., non-target strand, NTS) of the four simulated systems are shown in Fig. S4.

**Fig. 3:**
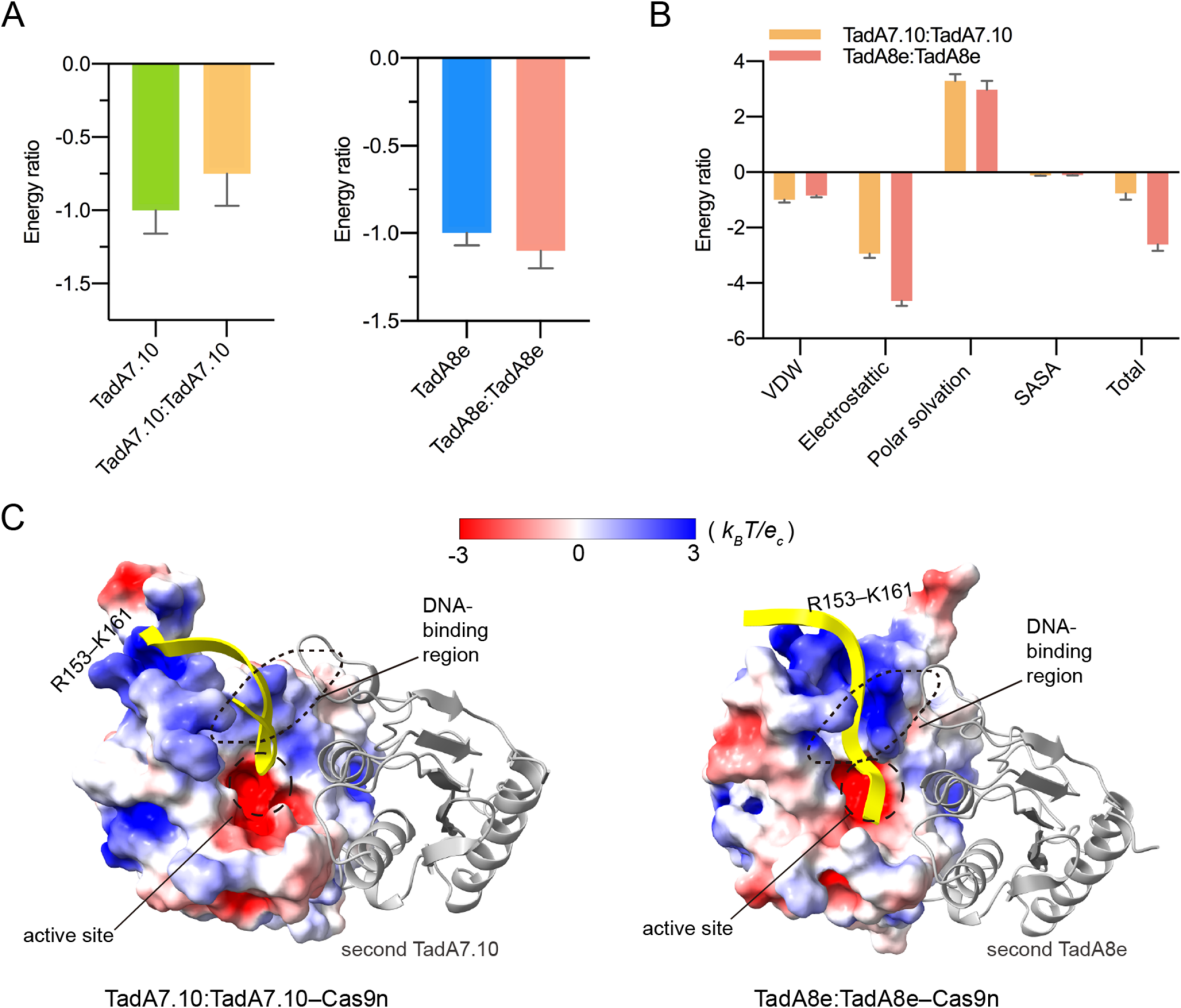
The binding energies and surface electrostatic potentials of the TadA-DNA complexes. (**A**) The binding energy ratios of TadA to DNA substrate, using the absolute values of TadA-Cas9n as the unit of 1. Data are presented as the mean ± SD. (**B**) The energy terms of TadA with the DNA substrate using the absolute values of the VDW energy of TadA7.10:TadA7.10 as the unit of 1. Data are presented as mean ± SD. (**C**) The surface electrostatic potentials of TadA calculated by APBS, on a scale from − 3 to 3 *k*_*B*_*T/e*_*c*_. Red represents negative electrostatic potential, blue represents positive electrostatic potential, and white is neutral

Given that the MM/PBSA calculation is well suited to ranking binding affinities that depend on relative values [43], we set the absolute energy value of TadA‒Cas9n as a unit of 1.0 and then converted the energies of the other systems in Fig. S4 into energy ratios of this unit. Using the dielectric constant 6 as an example, for TadA7.10 or TadA8e the energy ratios indicate that the binding affinities of TadA: TadA‒Cas9n and TadA‒Cas9n are comparable (Fig. 3A), suggesting that dimerization does not significantly affect the substrate binding. However, when comparing TadA7.10 with TadA8e, we found that the binding energies of TadA8e are 1.9 ~ 8.9 times those of TadA7.10 at all four dielectric constants (Fig. S4), strongly supporting that TadA8e has a higher DNA binding affinity for the DNA substrate than TadA7.10. Thus, TadA8e appears to be able to bind the DNA substrate in a monomeric form. Again, the eight-direction mutations also enhance the DNA binding capability of TadA8e, thereby enabling it to bind firmly to the substrate.

To determine the main forces driving the TadA8e binding to DNA, we further analysed the components of the binding energies. As shown in Fig. 3B and S5A, the binding is mainly driven by the electrostatic attractions. The improvement in the binding energy of TadA8e is attributed to the enhanced electrostatic attractions. In order to elucidate the structural basis for the difference in electrostatic interactions between TadA8e and TadA7.10, we calculated their surface electrostatic potentials using the APBS program [28].

As illustrated in Fig. 3C and S5B, the surface potentials indicate that the DNA binding region of TadA8e exhibits a higher positive charge density than that of TadA7.10. In TadA7.10, the loop comprising residues 157‒167 is far from the active site, whereas in TadA8e it is closer to the active site. The results in the segment of R153‒K161 upstream of this loop forming a continuous positively charged surface with the region around the active site, thereby increasing the positively charged area in the DNA binding region. Compared to TadA7.10, the electrostatic potential in the DNA binding region of TadA8e is more attractive to DNA, thereby increasing the binding affinity.

### Directed-evolution mutations increase positive charge density of DNA binding region

**Fig. 4:**
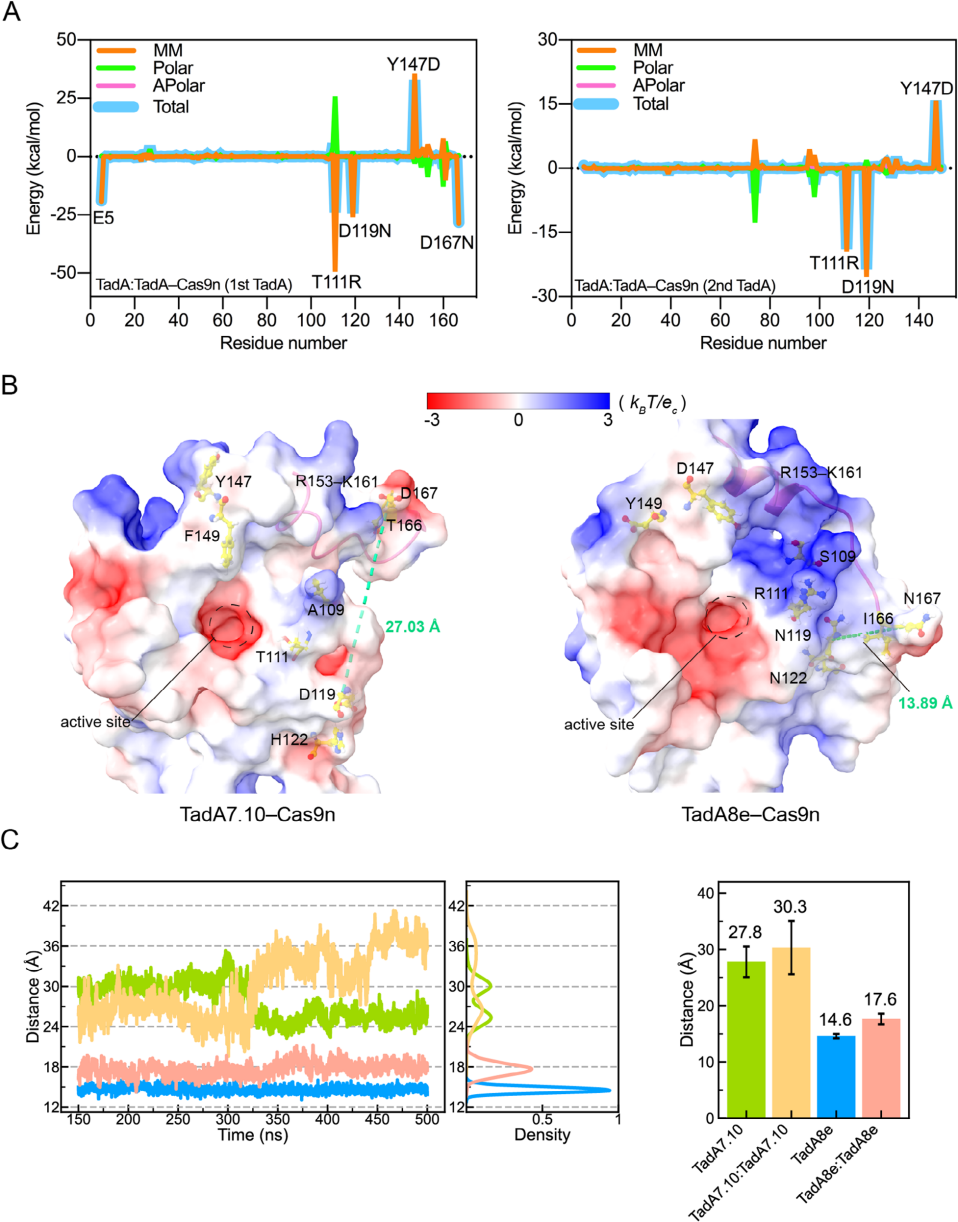
Identification of key mutations for the DNA binding. (**A**) ΔΔG per residue between TadA7.10 and TadA8e in the TadA: TadA‒Cas9n systems. (**B**) The surface electrostatic potentials of the mutations. Mutation residues are shown as yellow sticks. Red represents negative electrostatic potential, blue represents positive electrostatic potential, and white is neutral. The distances from the Cα atom of residue 119 to the Cα atom of residue 167 are shown as green dotted lines. (**C**) Time-dependent distances and corresponding distributions from the Cα atom of residue 119 to the Cα atom of residue 167

Given that TadA8e was obtained by mutating eight residues of TadA7.10, it can be reasonably assumed that the higher DNA-binding affinity of TadA8e is a consequence of these mutations. In order to quantitatively characterise the contributions of the mutations to the DNA binding, we calculated the binding energy difference (ΔΔG) for each amino acid between TadA7.10 and TadA8e. As an illustrative example, the results of the dielectric constant of 6 are presented in Fig. 4A and Fig. S6A. For the first TadA fused to Cas9n, T111R, D119N and D167N are the mutations with the most significant energy contributions (ΔΔG < -20 kcal/mol). Once again, electrostatic interactions are the main component of the energy contributions. Obviously, the mutations from D to N at both residues 119 and 167 eliminate the electrostatic repulsions to DNA; and the positively charged residue R111 could directly increase the electrostatic attractions to DNA (Table 1).

**Table 1:** Energy contributions of the eight directed-evolution mutations to the NTS binding (kcal⋅mol^− 1^)

Mutation	MM		Polar		Apolar			Total	
	TadA7.10	TadA8e	TadA7.10	TadA8e	TadA7.10		TadA8e	TadA7.10	TadA8e
A109S	-1.97	-1.34	1.01	0.73	-0.18		-0.04	-1.14	-0.65
T111R	-0.75	-44.40	0.27	20.27	-0.02		-0.30	-0.50	-24.43
D119N	25.23	-0.31	-0.89	0.07	0.00		0.00	24.34	-0.24
H122N	-0.08	-0.45	0.04	0.14	0.00		0.00	-0.05	-0.31
Y147D	-0.07	34.48	0.10	-3.38	-0.01		0.00	0.01	31.10
F149Y	-3.79	-4.28	0.22	2.56	-0.40		-0.31	-3.97	-2.03
T166I	0.11	-0.09	0.08	-0.01	0.00		0.00	0.19	-0.11
D167N	34.09	-0.27	-2.84	0.09	-0.04		0.00	31.20	-0.18

Similarly, for the second TadA in dimerization with the first, T111R and D119N also have the largest contributions (Fig. 4A). Somewhat differently, N119 in TadA8e contributes the most, probably due to the trans-dimerization of two TadA8e proteins, with the second N119 remaining close to N119 of the first TadA8e (Fig. S6B). Consequently, N119 in the second TadA8e is also in close proximity to the DNA substrate. And the mutation from D to N is beneficial in eliminating the repulsive electrostatic interactions. In contrast, R111 in the second TadA8e is slightly away from the DNA substrate and its energy contribution is slightly lower than that of the first TadA (Fig. S6B). It is likely that these mutations in both TadA8e proteins could enhance the DNA binding.

We next examined the effects of the mutations on the surface distributions of the electrostatic potentials of TadA8e. As shown in Fig. 4B, R111 in TadA8e increases the positively charged area within the DNA binding region, highlighting its key role in DNA binding. In TadA7.10, electrostatic repulsion between D119 and D167 results in the peptide of R153–K161 moving away from the active site, thereby disrupting the continuous surface of positive potentials (Fig. 4B). In TadA8e, D119 and D167 have been mutated to N, and the repulsions between them are eliminated, allowing N167 to approach N119. This then causes the peptide of R153–K161 and R111 to form a continuous surface of positive potentials, expanding the positively charged area in the DNA-binding region of TadA8e.

To further confirm that the distance from residues 119 to 167 in TadA7.10 is greater than that in TadA8e, we calculated their distances in TadA7.10 and TadA8e, respectively. As anticipated, the distances between residues 119 and 167 in the two simulated systems of TadA7.10 were 27.8 Å and 30.3 Å, respectively, which are considerably larger than those in corresponding systems of TadA8e (14.6 Å and 17.6 Å, respectively) (Fig. 4C), indicating a close correlation between these two residues in TadA8e.

### Experimental verifications of key mutations on binding and protein stability

To verify the key roles of T111R, D119N and D167N in enhancing the DNA-binding affinity, we constructed a TadA7.10 mutant with R111/N119/N167 and designated it as TadA7.10-3mut. Subsequently, the proteins of TadA7.10 and TadA7.10-3mut were expressed and purified, microscale thermophoresis (MST) measurements were performed to determine their DNA-binding affinities. Given that TadA acts only on ssDNA [23], to test whether the NTS in the structure 6VPC is a single-stranded DNA, we first predicted its secondary structure via the RNAStructure website (http://rna.urmc.rochester.edu/RNAstructureWeb/) [54], and found that the NTS may form a base-paired structure (Fig. S7A). So, five bases were mutated at either end of the NTS and obtained a 19-nt ssDNA (TTCTCTTCC*A*CTTTCTTTT) as the substrate used in the MST measurements. The error between the fluorescence intensities of all capillaries was found to be no greater than 10%, with a range of 1200–1320 (Fig. S7B). Data points outside this range were excluded.

**Fig. 5:**
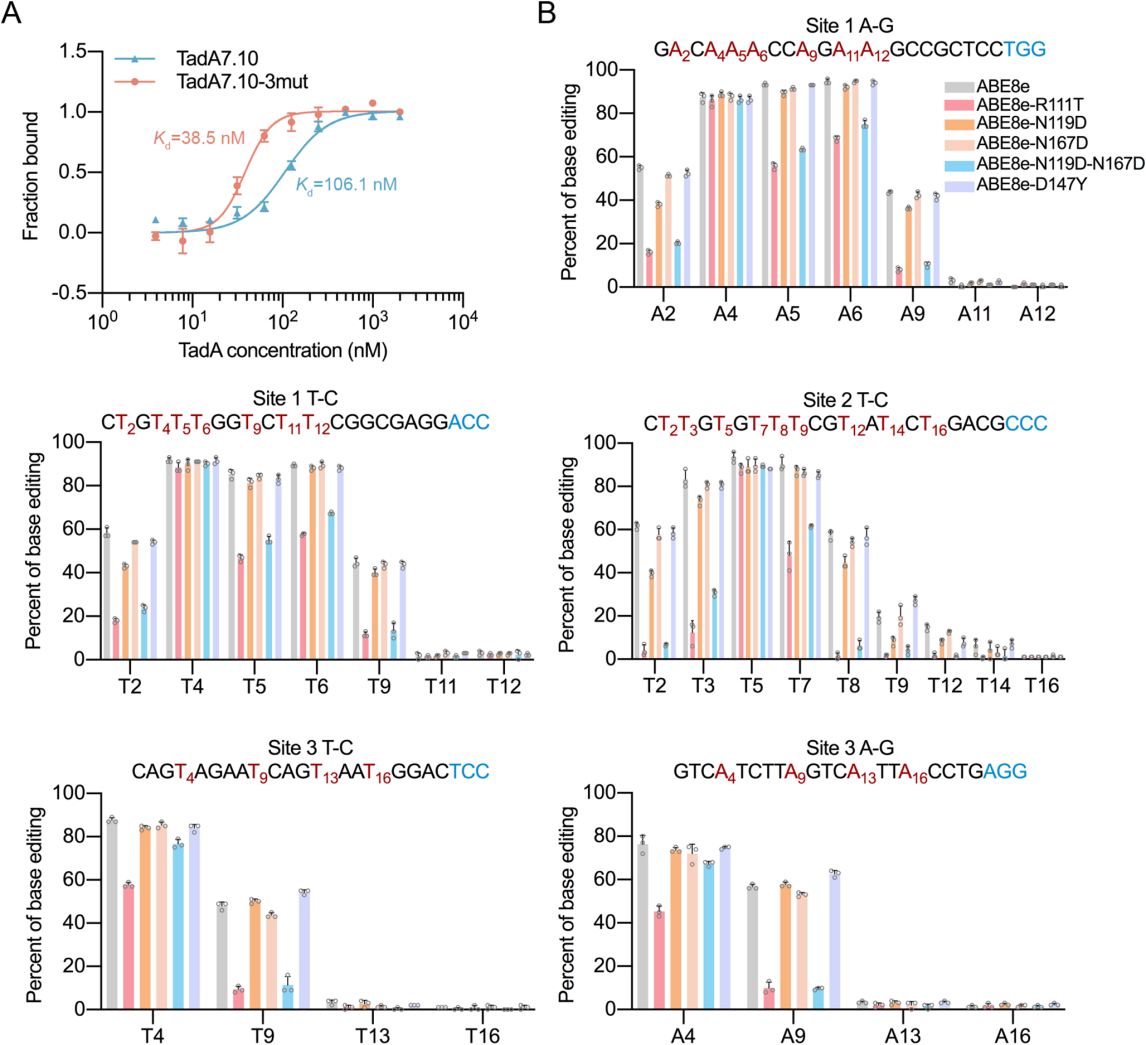
Experimental verification of the key mutations. (**A**) MST measurements for TadA7.10 and TadA7.10-3mut (R111/N119/N167). The K_d_ values of TadA7.10 and TadA7.10-3mut are 106.1 and 38.5 nM, respectively. Data are presented as mean ± SD of three independent experiments. (**B**) Base editing efficiencies for ABE8e, ABE8e-R111T, ABE8e-N119D, ABE8e-N167D, ABE8e-R111T-N119D and ABE8e-D147Y at three genomic sites in HEK293T cells. The targets As and Ts are shown in red, with a subscripted number indicating their relative position to the PAM (NGG PAM is counted as + 21 to + 23), and the PAM sequence is shown in blue. Editing efficiencies were analyzed by Sanger sequencing and EditR calculation. For all plots, dots represent individual biological replicates and bars represent the mean ± SD of three independent biological replicates

As shown in Fig. 5A, the MST results indicated that the equilibrium dissociation constants (*K*_d_) of TadA7.10 and TadA7.10-3mut were 106.1 and 38.5 nM, respectively. This suggests that the DNA-binding affinity of TadA7.10-3mut is approximately 2.8 times that of TadA7.10, which provides strong evidence that the residues R111/N119/N167 in TadA8e could increase its DNA-binding affinity. To further assess the DNA-binding effects of R111, N119 and N167 on editing activity, we performed an in vivo reversion mutation experiment. Four ABE8e variants were constructed as follows: ABE8e-R111T, ABE8e-N119D, ABE8e-N167D and ABE8e-N119D-N167D. Then, we used the generated mutants to target three gene loci in HEK293T cells, after which Sanger sequencing and EditR analysis were performed [55].

The reversion mutation results indicated a significant reduction in the activity of ABE8e-R111T (Fig. 5B), consistent with previous studies that its deamination rate is comparable to that of ABE7.10 [23]. Meanwhile, the activity of ABE8e-N167D is almost the same, and that of ABE8e-N119D decreased slightly (Fig. 5B). However, when both N119 and N167 were mutated back to those in ABE7.10, the activity of ABE8e-N119D-N167D decreased dramatically (Fig. 5B). These results suggest a coupled effect between these two residues, in agreement with our calculations. We also tested the effect of D147Y and found that the activity of ABE8e-D147Y was only minimally affected (Fig. 5B). Although the mutation form Y147 to D is energetically unfavourable, the single mutation appeared to have a limited effect on the activity, suggesting a potential coupling role with other mutations.

**Fig. 6:**
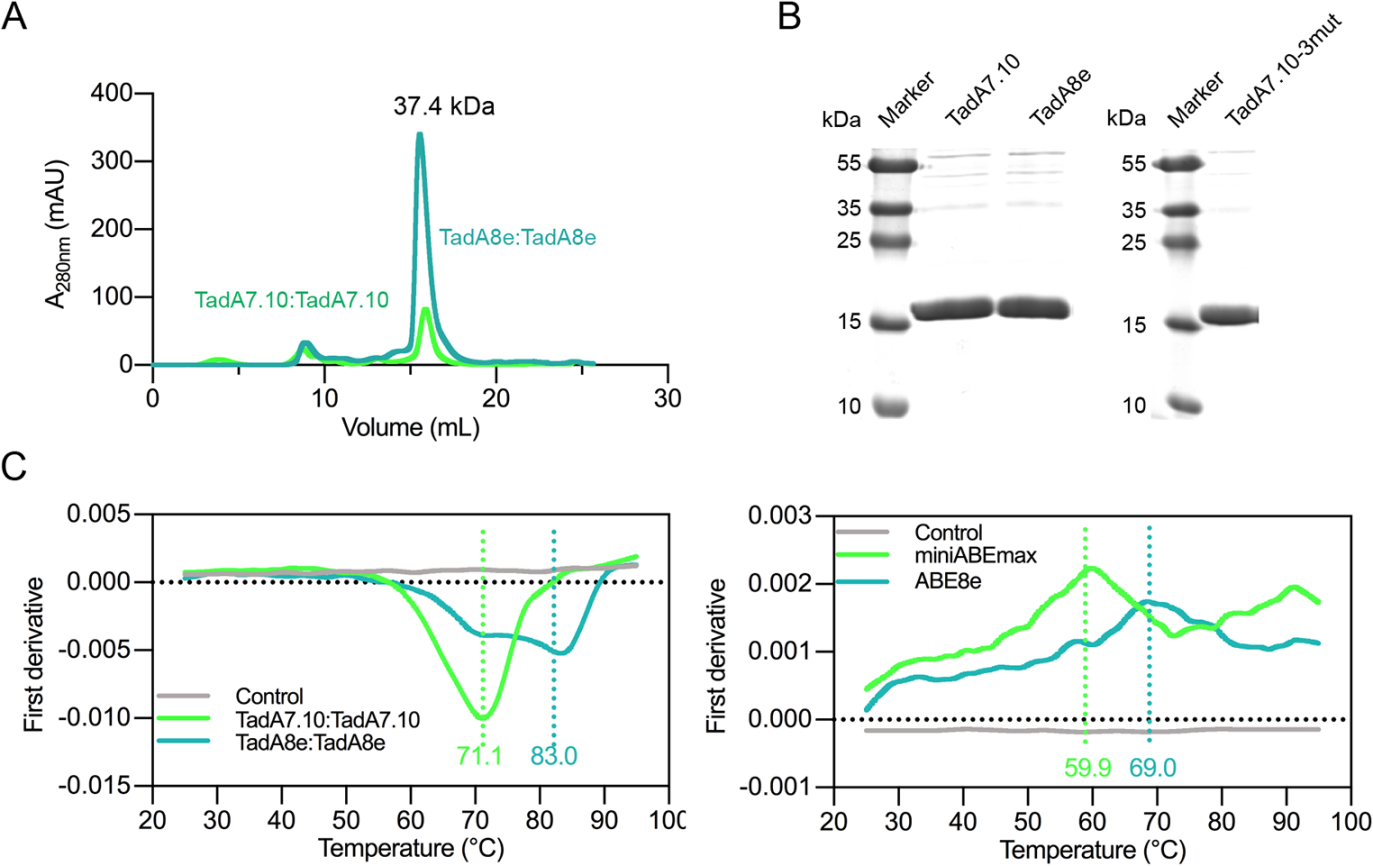
Thermal stability of TadA7.10, TadA8e, miniABEmax and ABE8e. **(A)** The UV absorption peaks of size exclusion chromatography for TadA7.10 and TadA8e. **(B)** The SDS-PAGE of TadA7.10, TadA8e and TadA7.10-3mut purified from *E. coli* Rosetta (DE3) cells. **(C)** First-order derivatives of the nano-differential scanning fluorimetry (nanoDSF) curves for TadA and ABE. The curves show that TadA7.10:TadA7.10 has a *T*_m_ of 71.1 °C, TadA8e: TadA8e has a *T*_m_ of 83.0 °C, miniABEmax has a *T*_*m*_ of 59.9 °C and ABE8e has a *T*_*m*_ of 69.0 °C

The computational results presented in Fig. 2 indicate that the mutations have an impact on the stability of TadA8e and ABE8e. To investigate this further, thermal shift assays were conducted using nanoscale differential scanning fluorimetry (nanoDSF) [44] to measure the melting temperatures (T_m_) of the purified TadA7.10, TadA8e, miniABEmax and ABE8e proteins, respectively. The UV absorption peaks of size exclusion chromatography indicated that the molecular weights of the TadA proteins were in the range of 44.0–29.0 kDa. Given that the molecular weight of the TadA monomer is approximately 18.7 kDa, this suggests that the TadA proteins form dimers (Fig. 6A-B). Thermal shift assays showed that the T_m_ of TadA8e is ~ 83.0 ^o^C, while that of TadA7.10 is about 71.1^o^C. Similarly, that of ABE8e is ~ 69.0 ^o^C, while that of miniABEmax is about ~ 59.9 ^o^C (Fig. 6C). Thus, the thermal stability of TadA8e and ABE8e is increased by ~ 12 ^o^C (T_m_) and ~ 9 ^o^C, respectively. This provides experimental evidence that TadA8e and ABE8e have higher thermostability than TadA7.10 and miniABEmax. Thus, we unexpectedly discovered that the eight directed-evolution mutations also significantly improved the thermal stability of TadA8e and ABE8e. This implies that the mutations also optimised the physicochemical properties of the proteins, which are also important for the enzymatic activity of ABE8e.

## Discussion

In this study, we combined MD simulations and experimental measurements to investigate the role of the eight directed-evolution mutations in the TadA8e deaminase. The MD simulations showed that TadA8e exhibits higher DNA-binding affinity than TadA7.10, and the electrostatic interactions were identified as the main driving force. The directed-evolution mutations were found to increase the positive charge density in the DNA-binding region of TadA8e, thereby increasing the electrostatic attractions with the NTS. Among the eight mutations, three were identified as key mutations (R111/N119/N167) for the DNA binding and verified by the experiments. Furthermore, we also discovered that the directed-evolution mutations significantly improved the thermal stability of TadA8e and ABE8e. Collectively, our data demonstrate that the base editing activities of ABEs are closely related to the DNA-binding affinity and protein stability.

As demonstrated by our simulations, TadA8e exhibits comparable DNA binding energy to the dimerised TadA8e: TadA8e, indicating that dimerisation may not be a prerequisite for binding to the DNA substrate. Consequently, removing dimerisation not only increases the number of functional ABEs, but also enhances the probability of targeting a site that is challenging to access by the dimerised TadA8e.The removal of of dimerisation could be a promising direction for future optimisation of ABEs.

Therefore, due to the enhanced DNA binding induced by the directed-evolution mutations, only one TadA8e deaminase is sufficient for ABE8e to attract the NTS of DNA to its active site. In addition to confirming the previously reported key mutation T111R [23], our study identified two other important amino acids that enhance substrate binding: N119 and N167. A number of studies have shown a positive correlation between the substrate-binding affinity and the nuclease activity [56–61]. For instance, the DNA-binding affinity and specificity of the zinc finger nucleases are the main determinants of their effective activity in human cells [57]. Similarly, the high affinity of activation-induced deaminase (AID) for DNA substrates is essential for its efficient deamination [60]. In accordance with the findings of our study, Rallapalli et al. also demonstrated that the D108N mutation of the early-evolved TadA enhances its early editing kinetics by increasing the substrate binding [25]. As we have pointed out, back mutation of the key DNA-binding residues R111/N119/N167 to the original residues dramatically reduced the editing activity of ABE8e. All these results suggest that the high affinity of TadA8e for the target DNA may be an important determinant of its base editing activity.

Certainly, further studies are needed to elucidate the relationship between the DNA-binding affinity and the base editing activity. For example, a direct and precise quantification of the contribution of DNA binding affinity to the base editing activity of ABE8e is still lacking, which limits our comprehensive understanding of the ABE8e activity mechanism by the targeted mutations. Meanwhile, as also shown in a previous study [23], TadA8e is a multi-turnover enzyme, i.e., after deaminating an adenosine substrate, it immediately dissociates from it and then binds to a new adenosine for the next round of deamination. Consequently, thermodynamically stable TadA8e-substrate complexes are not formed. Therefore, it has been difficult to conduct reliable MST experiments to measure the binding affinity of TadA8e for the substrate. Undoubtedly, new experimental approaches are needed for future functional and mechanistic studies of ABE8e. In addition, further computational studies to predict new mutations that may improve binding affinity will also be an interesting extension of this work.

In conclusion, ABEs are important molecular machines for precise genome editing. Directed evolution has been an important approach to their optimisation. Therefore, it is very important to elucidate the molecular origin of the directed-evolution mutations in order to further optimise ABEs. Our study elucidates the functional consequences of the eight directed-evolution mutations in the base editing catalysis of ABE8e. We demonstrate that these mutations increase the DNA-binding affinity and protein stability, which contribute to the improved base editing activity of ABE8e. Our findings provide new insights into the optimisation of BEs, highlight the importance of understanding the molecular mechanisms underlying directed evolution, and thus provide a rational basis for further catalytic optimisation of the system for higher efficiency and specificity. Indeed, higher editing efficiency allows for a reduction in the required dose of enzyme for gene therapy, thereby reducing the potential side-effects and improving the economic feasibility of treatment. Furthermore, efficient base editors are powerful tools for studying gene function, leading to the discovery of new diagnostic markers or therapeutic targets. In addition, efficient base editors are more precise and faster than conventional breeding methods, which promises to accelerate the breeding of new species adapted to environmental challenges.

### Electronic supplementary material

Below is the link to the electronic supplementary material.

Supplementary Material 1

## Data Availability

All data needed to evaluate the conclusions in the paper are present in the paper and/or the Supplementary information. Additional data related to this paper may be requested from the authors.

## Abbreviations

ABEs: Adenine base editors
CRISPR: Clustered regularly interspaced short palindromic repeats
Cas: CRISPR-associated
DSBs: Double-stranded breaks
NHEJ: Non-homologous end joining
Indels: Insertion or deletion mutations
Cas9n: Cas9 nickase
ssDNA: Single-stranded DNA
sgRNA: Single guide RNA
TS: Target strand
NTS: Non-target strand
CBEs: Cytosine base editors
ABEs: Adenine base editors
TadA: tRNA adenosine deaminase
Cryo-EM: Cryo-electron microscopy
T_m_: Melting temperature
MD: Molecular dynamics
RMSDs: Root mean square deviations
Rg: Radius of gyration
MST: Microscale thermophoresis
*K*_d_: Equilibrium dissociation constant
