# Supplementary material for: Directed-evolution mutations enhance DNA-binding affinity and protein stability of the adenine base editor ABE8e
Source: Cell Mol Life Sci. 2024 Jun 14;81(1):257. doi: 10.1007/s00018-024-05263-7 (PMC11335294; doi:10.1007/s00018-024-05263-7)
Supplement: Supplementary file 1 — Supplementary Material 1 [file 18_2024_5263_MOESM1_ESM.pdf]

# Supplementary Information

## Directed-evolution mutations enhance DNA-binding affinity and protein stability of the adenine base editor ABE8e

Haixia Zhu[1], Lei Wang[1], Ying Wang[1], Xinyi Jiang[1], Qin Qin[1], Menghua Song[1], Qiang Huang[1, 2]*

[1]*State Key Laboratory of Genetic Engineering, Shanghai Engineering Research Center of Industrial Microorganisms, MOE Engineering Research Center of Gene Technology, School of Life Sciences, Fudan University, Shanghai 200438, China*

[2]*Multiscale Research Institute of Complex Systems, Fudan University, Shanghai 201203, China*

*Corresponding author at: School of Life Sciences, Fudan University, Shanghai 200438, China. Email: huangqiang@fudan.edu.cn

**Contents:**

Supplementary Figures 1-7

Supplementary Tables 1-3

## Supplementary Figures

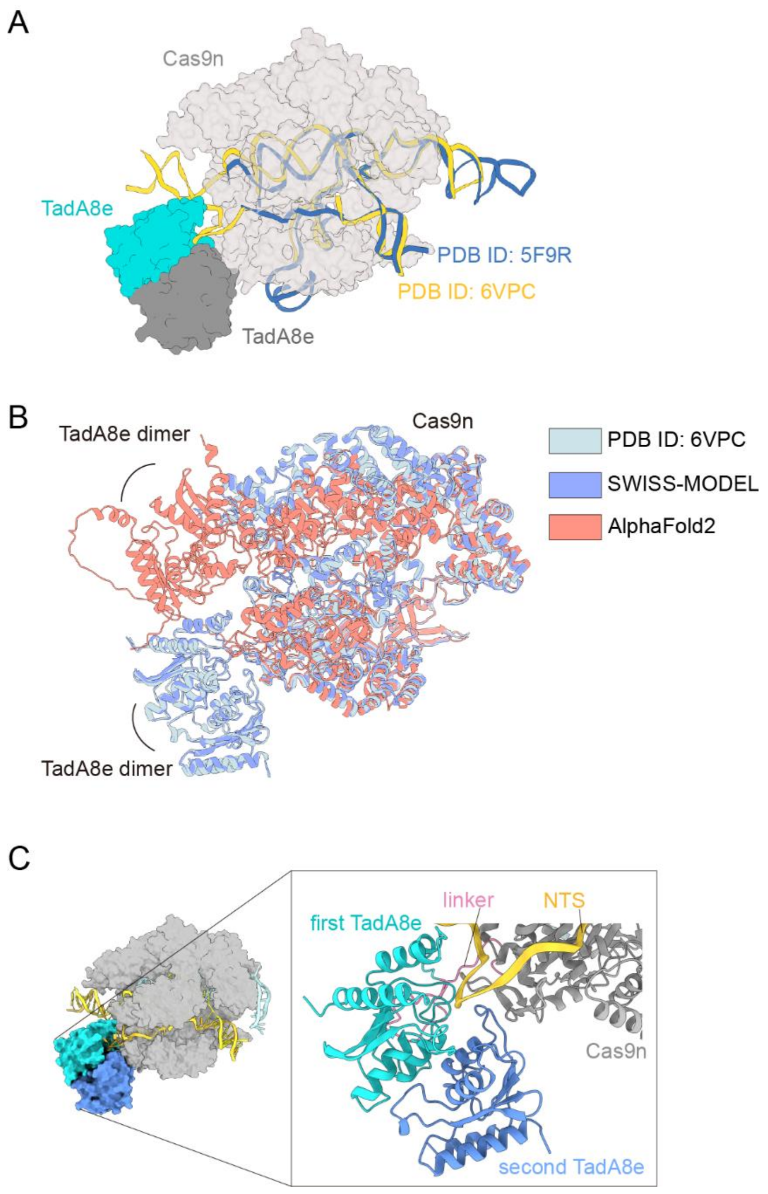


**Figure S1. The full-length ABE8e model.** (A) The sgRNA–DNA backbone superimposition of ABE8e structure (PDB ID: 6VPC, orange) onto the SpCas9 structure (PDB ID: 5F9R, dark blue). (B) Superposition of the SWISS-MODEL model (purple) and the AlphaFold2 model (red) of TadA8e:TadA8e–Cas9n onto PDB ID: 6VPC (cyan). (C) Domain architecture of ABE8e: SpCas9 (grey), RNA (cyan), NTS (yellow), linker (pink), and TadA8e dimer (light and dark blue).

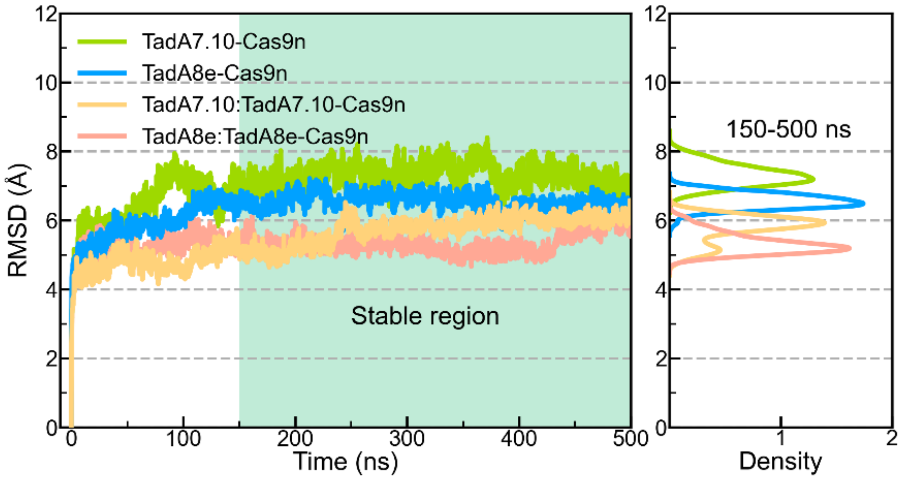


**Figure S2. Time-dependent RMSDs of the ABE structures in the MD simulations.** Four systems have been equilibrated after about 150-ns simulations.

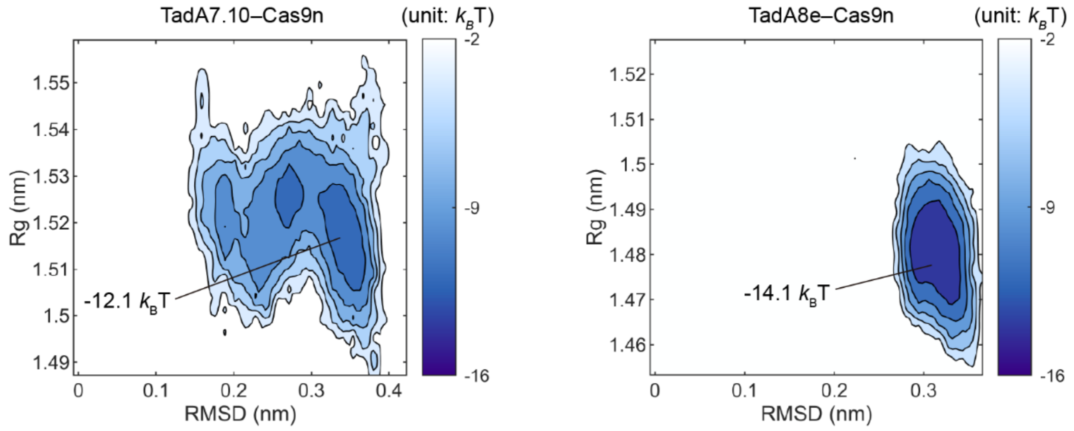


**Figure S3. The conformational stability of TadA7.10 and TadA8e.** Free energy landscapes of the TadAs projected onto the RMSD of the protein backbone atoms and the radius of gyration (Rg), where $k_B$ is the Boltzmann constant and $T$ is the simulation temperature (310 K).

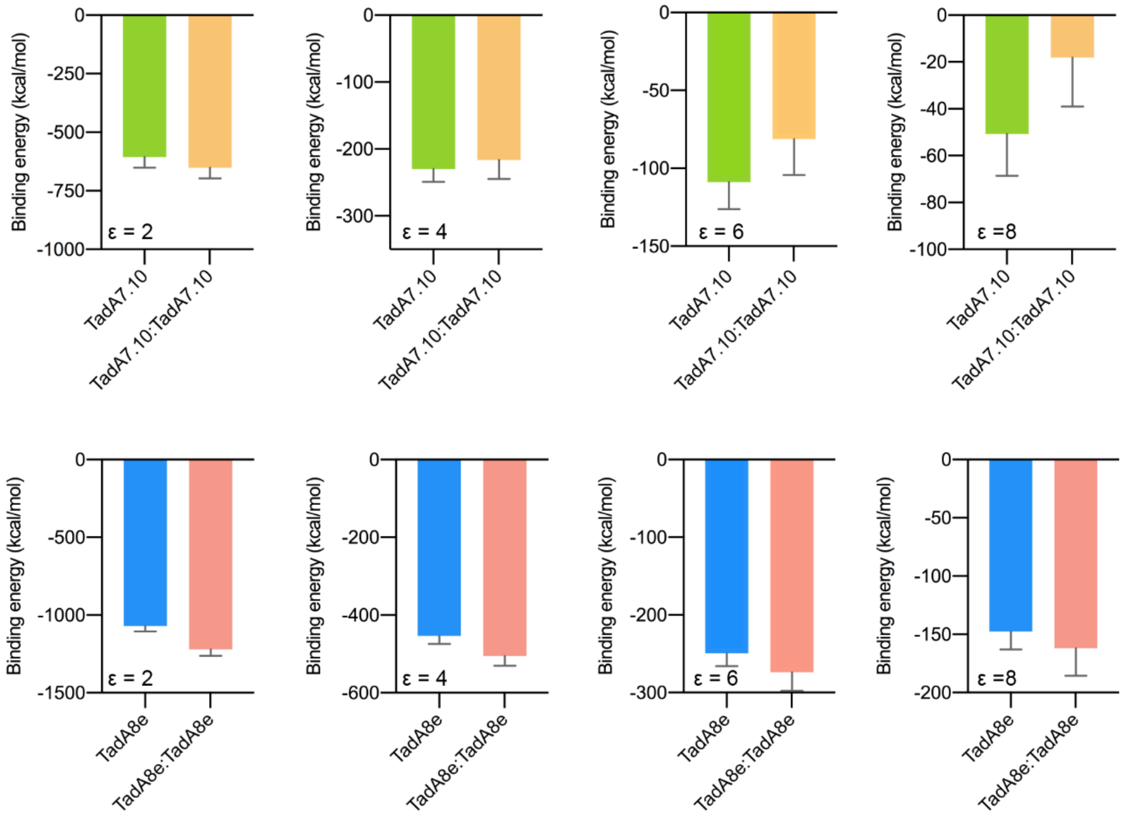


**Figure S4. The binding energies of TadA to DNA substrate.** The binding energies of TadA to the DNA substrate in four simulated systems at four different dielectric constants. Data are presented as the mean ± SD.

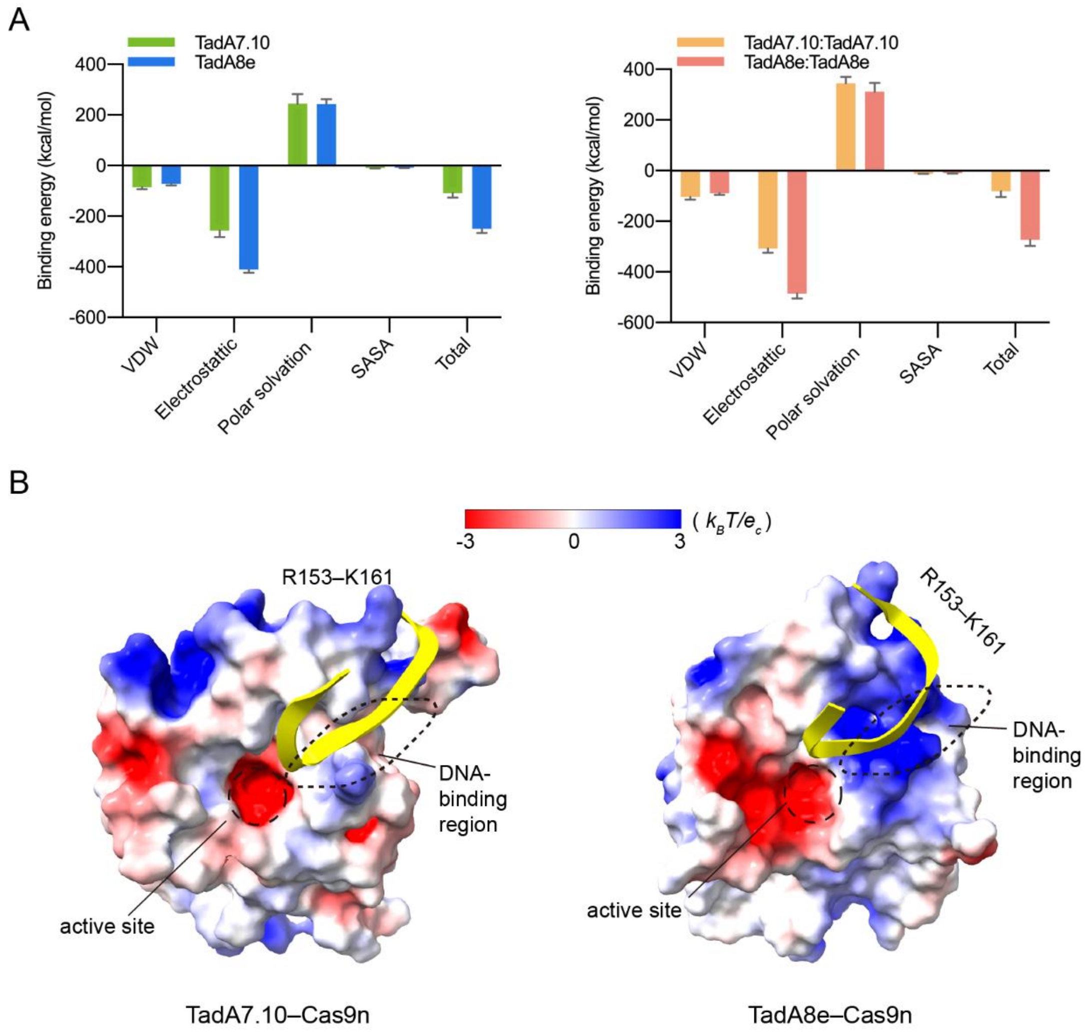


**Figure S5. Energy terms and surface electrostatic potentials of TadA–DNA complexes.** (A) Binding energy terms of TadA with the DNA substrate at a dielectric constant of 6. Data are presented as the mean ± SD. (B) The surface electrostatic potentials of TadA in simulated TadA–Cas9n systems, calculated by APBS on a scale from -3 to 3 $k_BT/e_c$. Red represents negative potential, blue represents positive potential, and white is neutral.

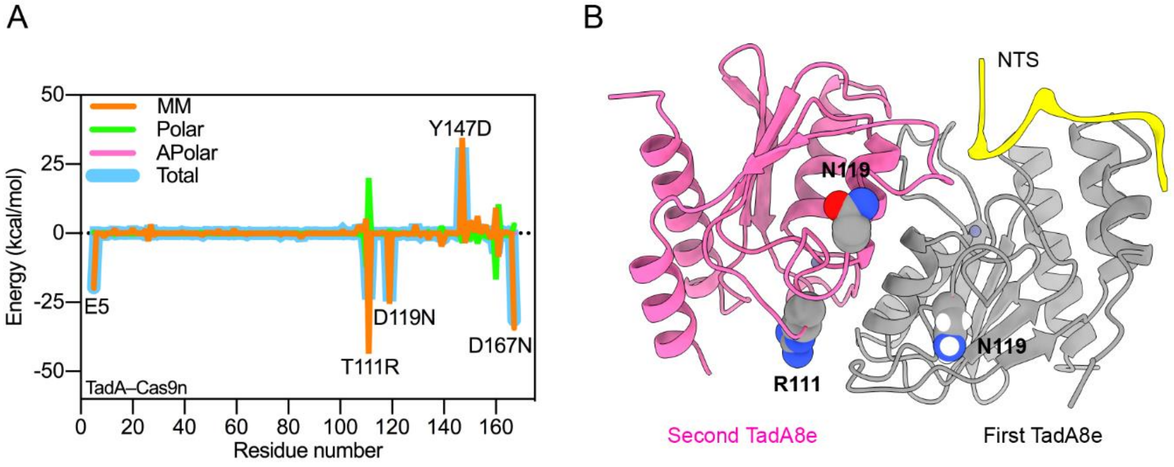


**Figure S6. Energy contributions of mutations and their positions.** (A) ΔΔG per residue between TadA8e and TadA7.10 in the TadA–Cas9n system. (B) The positions of N119 and R111 in ABE8e (PDB ID: 6VPC), R111 and N119 are shown as spheres.

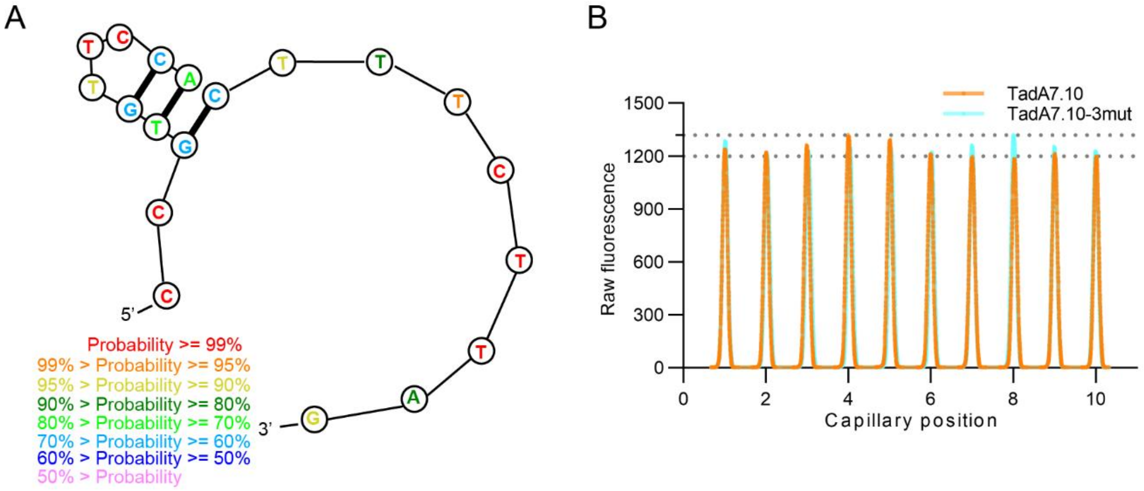


**Figure S7. Sample and screening conditions for MST measurements.** (A) The secondary structure of the NTS in ABE8e (PDB ID: 6VPC) predicted by the RNAStructure website at http://rna.urmc.rochester.edu/RNAstructureWeb/. (B) The errors between the fluorescence intensities of all capillaries are within 10%, i.e., 1200-1320.

## Supplementary Tables

**Table S1.** Protospacer sequences for mammalian genomic sites.

| Site | Protospacer | PAM |
|---|---|---|
| 1 | GACAAACCAGAAGCCGCTCC | TGG |
| 2 | GAACACAAAGCATAGACTGC | GGG |
| 3 | GTCATCTTAGTCATTACCTG | AGG |

**Table S2.** Primers used for amplification of genomic DNA of mammalian cells.

| Site | Forward primer | Reverse primer |
|---|---|---|
| 1 | AGCCCTCTTTTTATTGGAACTGTG | CCGACTGGTCCACTTACCTA |
| 2 | TGAATGGATTCCTTGGAAACAATGA | CCAGCCCCATCTGTCAAACT |
| 3 | AACGGAACTCAACCATTAAGCA | CCAACATACAGAAGTCAGGAATGC |

**Table S3.** Amplicons for Sanger sequencing analysis.

| Site | Amplicon |
|---|---|
| 1 | CCGACTGGTCCACTTACCTATCACAATCACAACTGCAATATTACATAAATAGTCTGTTAGAGAAG<br>GACAACTGAATCTGCAAATCAGTATCTTCTCAATGAGAGTACAAGGAGGCAATGGCTACATACGA<br>TGGACAAACCAGAAGCCGCTCCTGGGCTATGTTTACTATTTACTTTTATGGTATAAAAATGTTCT<br>CAGTCAAGCAGTTTCAACAATAGATACCACAGTTCCAATAAAAAGAGGGCT |
| 2 | TGAATGGATTCCTTGGAAACAATGATAACAAGACCTGGCTGAGCTAACTGTGACAGCATGTGGTA<br>ATTTTCCAGCCCGCTGGCCCTGTAAAGGAAACTGGAACACAAAGCATAGACTGCGGGGCGGGCCA<br>GCCTGAATAGCTGCAAACAAGTGCAGAATATCTGATGATGTCATACGCAC<br>AGTTTGACAGATGGGGCTGG |
| 3 | CCAACATACAGAAGTCAGGAATGCTTGAATATAAATTTATTATTACTCTATGTTCTATTTAAGTT<br>TTCATGTTCTAAAAATGTATCCCAGTTTACACGTCTCATATGCCCCTTGGCAGTCATCTTAGTCA<br>TTACCTGAGGTGTTCGTTGTAACTCATATAAACTGAGTTCCCATGTTTTGCTTAATGGTTGAGTT<br>CCGTT |
